# Unexpected allergic reactions to food, a prospective study

**DOI:** 10.1186/2045-7022-3-S3-P143

**Published:** 2013-07-25

**Authors:** A Michelsen, HV Os-Medendorp, A Versluis, A Kruizinga, J Castenmiller, H Noteborn, G Houben, A Knulst

**Affiliations:** 1 Dermatology and Allergology, UMC Utrecht, Utrecht, the Netherlands; 2 Netherlands Organisation for Applied Scientific Research, Zeist, the Netherlands; 3 Dutch Food and Consumer Product Safety Authority, Utrecht, the Netherlands

## Background

Severe reactions and even fatalities occur in patients with food allergy, but frequency data are scarce. The aim of this study is to investigate the frequency, severity and causes of unexpected allergic reactions to food in adults diagnosed with food allergy.

## Methods

A prospective cohort study will be carried out from January 2012 to June 2014. A total of 250 patients with a physician-confirmed diagnosis of food allergy will be included and followed during one year. Outcome measures are frequency, severity and causes of unexpected reactions. Participants complete an online questionnaire after every assumed accidental exposures and send in the product with food label they reacted on. The product will be analyzed to detect if the suspected allergens are present.

## Results

In November 2012 58 patients have been included and 32 unexpected reactions were reported. Preliminary results show that 15 of the unexpected reactions were caused by pre-packaged products.

Only seven of the 15 patients read the label well. In four of these seven products, the labels did not mention the food allergen or indicate possible cross-contamination. Another 6 reactions were caused by using products without a label. Only two patients checked the ingredients by asking the seller. Two reactions took place abroad.

Up to now all unexpected reactions on composite meals (n=9) took place outdoors, for example at a friends’ or relatives home, at school or in a restaurant. Fourteen products were sent in to analyze the presence and amount of allergens; analyses are underway.

Six patients reported more than once an unexpected reaction.

Updated data will be presented at the congress.

## Conclusion

This prospective study will give more reliable insight in the frequency, severity and causes of unexpected allergic reactions. Results of this study will attribute to optimization of strategies to support patients in dealing with their food allergy; to prevent unexpected reactions by increasing awareness and knowledge in food industry, among retail and restaurants staff.

## Disclosure of interest

A Michelsen: Grant/research support from the Dutch Food and Consumer Product Safety Authority, H Os-Medendorp: None declared, A Versluis: None declared, A Kruizinga: None declared, J Castenmiller: None declared, H Noteborn: None declared, G Houben: None declared, A Knulst: None declared.

